# Synthesis and evaluation of composite TiO_2_@ZnO quantum dots on hybrid nanostructure perovskite solar cell

**DOI:** 10.1038/s41598-022-13903-w

**Published:** 2022-06-14

**Authors:** Negin Pezhooli, Jamal Rahimi, Farzam Hasti, Ali Maleki

**Affiliations:** 1 Catalysts and Organic Synthesis Research Laboratory, Department of Chemistry, Iran University of Science and Technology, Tehran, 16846-13114 Iran; 2 Department of Environmental Sciences, Gorgan University of Agricultural Sciences and Natural Resources, Gorgan, Iran

**Keywords:** Energy, Environmental social sciences

## Abstract

This research is an interdisciplinary study aimed at helping the environment and producing clean energy. Therefore, one of the goals of this research towards the field of nanotechnology is the application of nanotechnology in the preparation of solar cells and the provision of optimal and efficient cells. Perovskite solar cells are of particular importance because of the high efficiencies that they have achieved in recent years. The use of quantum dots has also played an important role in the efficiency of these cells and their efficiency. The TiO_2_@ZnO nanocomposite was selected and synthesized for this study. The application of this nanocomposite with different ratios of TiO_2_ and ZnO quantum dots was investigated and their efficiency was determined. Although the efficiency of the fabricated cell was reported to be about 5% in a solar cell made of TiO_2_@ZnO composite, the efficiency can be increased by optimizing conditions such as the optimal location for these cells or by compositing with other materials.

## Introduction

With the widespread use of fossil fuels around the world, energy shortages have become a serious challenge. To solve these problems, the use of renewable energy such as solar energy has been studied^1–3^. Sun is a huge source of energy and solar energy is the unique renewable, convenient, and main source of all the available energy on Earth. Many efforts have been made today to exploit this great energy source. Solar cells are one way to use this energy. Nowadays, organometallic perovskite solar cells have become a powerful alternative to popular commercialized silicon solar cells due to their easy synthesis, inexpensive components, and low fabrication costs^4,5^.

In the past few decades, research activities on nanomaterials have grown rapidly since materials in nanosize exhibit completely different properties as compared to their bulk properties^6^. Quantum dots (QDs) grabbed lots of attention in recent years because of their amazing characteristics in optics. The energy gap of QDs depends on their sizes and synthesis methods^7–10^. Quantum dots (QDs) have the advantage of tunable bandgap as a result of size variation as well as the formation of intermediate bands^11^.

Since the application of perovskite light absorber with the formula (CH_3_NH_3_PbI_3_) in the construction of solid-state solar cells in 2012, a lot of research work has been done in the field of perovskite solar cells, so that in a few years the efficiency of these cells to increase by 25.5%^12–14^. Studies show that the research into improving the efficiency of solar cells includes introducing new compounds or new methods of preparing these cells or improving existing compounds and methods. Since the solid-state perovskite cell consists of several layers consisting of compact TiO_2_, mesoporous TiO_2_, perovskite light-absorbing layer, a hole-transport layer, and electrode (Au)^15^, these studies can be performed on any part of the cell^16^. Nanotechnology and nanoscale materials can be introduced in some of these layers to increase the efficiency of the solar cell. To review some of the research on the application of nanotechnology in the field of perovskite solar cells, the following can be referred to: A: Research cavity barrier layer^17^, B: Mesoporous TiO_2_ research layer^18,19^, C: Perovskite light-absorbing layer of research^20,21^, D: study hole transfer layer^22,23^.

Increase energy efficiency, can lead to the optimization of perovskite solar cell construction conditions, which can be used in the field of nanotechnology. Also, locating and optimizing the establishment of solar cell farms is another way to increase energy efficiency.

This research is an interdisciplinary study that aims to help the environment and produce clean energy, so one of the objectives of this research in the field of nanotechnology and the environment is the application of nanotechnology in the preparation of solar cells and the presentation of optimal cells and it is productive. In this regard, as an example, we will synthesize the TiO_2_@ZnO composite and study the electron donor properties, improve the gap band and material absorption coefficient, and its effect on the efficiency and flow-potential diagram in the perovskite solar cell. This article has been prepared in two parts. The second part of this research has discussed suitable locations for perovskite solar cells in Kurdistan province located in western Iran^24^.

### Background on nanocomposite and perovskite solar cell

One of the goals of this research in the field of nanotechnology is to provide optimal and efficient solar cells. As an example, we will synthesize the TiO_2_@ZnO composite and study its electron-giving properties in the perovskite solar cell. To do this, we used the quantum dots of TiO_2_ and ZnO. Quantum dots are semiconductor crystals in the nanoscale (1–10 nm). Semiconductors are materials whose electrical conductivity is the interface between the electrical conductivity of conductive and non-conductive materials. Titanium dioxide due to its many properties including high refractive index, Lewis acid property, semiconductor and as an absorber of ultraviolet and visible light in photocatalytic applications and cheapness, high chemical stability, and non-toxic nature among other important metal oxides has special importance. Zinc oxide is also an important semiconductor with a band gap of 3.37 electron volts and high excitation energy of 60 mV at room temperature. In recent years, zinc oxide has attracted much attention mainly due to its unique optical, electronic, and piezoelectric properties, as well as its potential application in solar cells, blue light emitting diodes, sensors, and dimmers. This material can be used in optical instruments in ultraviolet and visible regions. At the nanoscale, it is also a highly transparent semiconductor with strong luminescence at room temperature, making it an ideal choice for a variety of sensors, laser diodes, displays, and transparent electrodes. Zinc oxide is biocompatible and safe and can be used in medicine and solar cells. Composite refers to solids that have more than one component used in their structure. A composite material is a physical, not a chemical, mixture on a macroscopic scale of two or more different materials. These materials retain their physical and chemical properties, but in general, the mixture has better properties than each of its components^25^. The bonds that small material makes with its surrounding phases are much stronger than on larger scales. Accordingly, a new branch of composites called nanocomposites has been introduced and developed. The nanocomposite is a composite material in which at least one of its constituent phases has nano dimensions (between 1 and 100 nm)^26^. Nanocomposites have a wider range of applications compared to other composites due to their more desirable physical, mechanical, and chemical properties^27^. In general, there are three generations of solar cells, of which the perovskite solar cell is a third generation solar cell. The general formula for perovskite compounds is ABX_3_, in which (A) can be a mono-valent organic or inorganic cation surrounded by twelve anions. (B) Is a divalent cation of the group of four principal bonds with six anions that forms an octagon. The combination of these octahedrons has created cavities that are occupied by a cation of organic or inorganic capacity and a perovskite structure. And X represents halogen atoms. The total oxidation number of cations and anions is equal to three, which has contributed to the charge balance and stability of the crystal structure^28^. Synthesis of the MAPbI_3_ compound performed by Weber and proposed a cubic structure for this compound and showed that the methyl ammonium group or C_3_v symmetry must be twisted to provide the necessary symmetry to form the facet octagon. As mentioned, in these compounds, phase change occurs with temperature change, and the combination, MAPbI_3_, also changes phase with temperature change from cubic to tetragonal. In addition to sensitivity to temperature and pressure, perovskite compounds are highly sensitive to moisture, which should be taken into account when preparing these compounds, so that they are not exposed to moisture because water enters the crystal structure of these compounds. These compounds change from black with the formula MAPbI_3_ to yellow with the formula MA_4_PbI_6_·2H_2_O^29^. The results of the research indicate the sensitivity of these compounds to temperature, pressure, and humidity, and this evidence suggests that when preparing these compounds, temperature and humidity control should be considered to obtain a perovskite compound with the appropriate phase.

## Materials and methods

### Laboratory equipment and chemicals

#### Laboratory equipment

The products were identified using infrared spectra by Shimadzu infrared spectrometers in the form of potassium bromide tablets. FE-SEM (Field Emission Scanning Electron Microscope) images were taken using the MIRA3 TESCAN device.

#### Chemicals

All the chemicals Zinc acetate dihydrate (Zn(CH_3_COO)_2_·2H_2_O, 99%), Potassium hydroxide (KOH, 99%), Titanium isopropoxide (C1_2_H_2_8O_4_Ti, 98%), methylamine (CH_3_NH_2_ (≥ 98%), hydroiodic acid (HI, 57%), Lead(II) iodide (PbI_2,_ 99%), hydrochloric acid (HCl, 37%) and solvents required in the tests such as ethanol (C_2_H_6_O, 96%), water deionizer, diethyl ether ((C_2_H_5_)_2_O, 99.5%), Isopropanol (C_3_H_8_O, 99%), dry ethanol 99%), ethyl acetate (C_4_H_8_O_2,_ 99%) were purchased from Sigma-Aldrich and Merck sources.

### Synthesis of materials

#### Synthesis of ZnO quantum dots

They were first agitated by a magnetic stirrer in various containers of 5049/0 g KOH in 30 ml ethanol and 0.03294 g Zn(CH_3_COO)_2_·2H_2_O in 150 ml ethanol, and then ultrasonic at room temperature for one hour. Then drop by drop of a container containing KOH solution was added to the container containing the solution of acetate for two hours by magnetic stirring at room temperature.

5 ml ethyl acetate was then added to obtain ZnO quantum dot precipitate. Finally, the resulting solution was washed with additional ethanol (added ethanol and passed through filter paper for several times) and placed in an oven at 70 °C to dry^30^.

#### *Synthesis of TiO*_*2*_* quantum dots*

12 ml titanium isopropoxide (C_12_H_28_O_4_Ti) was added to 20 ml ethanol and stirred for 20 min at room temperature to form a precursor solution with a magnetic stirrer. Then aqueous ethanol solution (ethanol/water 1:1) was added drop by drop to the precursor solution under ultrasound and then the solution was stirred with a magnetic stirrer for one hour at room temperature. Finally, the whole solution was placed in an autoclave at 150 °C for 12 h.

The resulting precipitate was cooled to room temperature and then centrifuged. The solution was then washed with water and ethanol (added mixture of ethanol and water and passed through filter paper for several times), and placed overnight at 50 °C to dry. The precipitate was then collected and calcined at 45 °C for 2 h^31^.

#### *Synthesis of CH*_*3*_*NH*_*3*_*I*

30 ml methylamine was stirred with 32.3 ml of the hydriodic acid in a 250 ml round bottom flask at 0 °C (ice bath) for 2 h. The solution was then rotated at 50 °C to remove solvents, which precipitated. The crude white to slightly yellow CH_3_NH_3_I product was washed three times with diethyl ether and passed through filter paper for several times to give a white powder. After filtration, the solid was collected and dried in a vacuum oven at 60 °C for 24 h. The resulting white solid was used without purification^32^.

#### *Synthesis of CH*_*3*_*NH*_*3*_*PbI*_*3*_* nanoparticles*

To obtain CH_3_NH_3_PbI_3_ nanoparticles, two compounds, CH_3_NH_3_I and PbI_2_, were each separately dissolved in isopropanol. The manufacturing steps are as follows: First, 8 ml CH_3_NH_3_I (0.25 M) solution was sonicated for 30 s. Then 6 ml PbI_2_ (0.25 M) solution was added dropwise to CH_3_NH_3_I solution for 30 min. A color change from yellow to dark brown was observed after the addition of two precursors and their mixing, indicating the occurrence of a chemical reaction. In this experiment, the purity of PbI_2_ is very important because if it is not pure enough, a good result will not be obtained^33^.

#### *Composite synthesis of TiO*_*2*_*@ZnO quantum dots*

In the synthesis of this composite, different amounts of TiO_2_ with ratios of 1:1, 2:1, and 1:2 to the quantum dots of ZnO were used. In a 1:1 ratio in separate containers, 0.084 g KOH in 5 ml ethanol and 0.055 g zinc acetate along with 0.055 g quantum dots of TiO_2_ in 25 ml ethanol was first stirred with a glass stirrer and sonicated at room temperature for one hour. Then drop by drop of KOH-containing solution was added to the zinc-containing solution for 2 h by magnetic stirring at room temperature, and then 3 ml ethyl acetate was added to precipitate ZnO quantum dots. The resulting solution was washed with excess ethanol and water and placed at 70 °C to dry. A 2:1 ratio (0.1 g TiO_2_, 0.055 g ZnO) and a 1:2 ratio (0.1 g TiO_2_, ZnO) of TiO_2_ and ZnO were used in the same manner as previously. Finally, the perovskite solar cell was fabricated by spin coating deposition and immersion^21,34,35^.

#### Synthesis of perovskite solar cell

All the following steps have been performed on the FTO conductive glass after etching and washing.

The following method was used to prepare a compact layer cell with the spin coating method. Two solutions were prepared in two glass vials as a combination of (1) 1.14 ml tetra isopropyl ortho-titanate (TTIP) and 10.12 ml ethanol, (2) 0.14 ml hydrochloride and 10.12 ml ethanol. The ethanol consumed is as waterless as possible, otherwise, the solution is not clear and milky or cloudy. Solution No. 2 was added drop by drop to solution No. 1, and the final solution was stirred with a magnetic stirrer for about 1 h. The solution was then filtered through a 220 nm PTFE filter.

To make the TiO_2_ compression layer, we used a spin coating device at 2000 rpm for 30 s. The amount of solution should be such that it covers the entire surface of the substrate.

Titanium dioxide and ethanol paste in different proportions are used to prepare the suspension of the mesoporous layer TiO_2_ layer. These two substances are mixed in ratios of 1:3.5 and we used a spin coating device at 5000 rpm for 30 s. When cooled to a temperature of 70 °C, the layers were removed from the oven and placed on a hot plate. (During perovskite deposition, the layers should be at 70 °C.) Then PbI_2_ solution was mixed with CH_3_NH_3_I solution in a 1:1 ratio and stirred for 2 h at 70 °C. The solution was coated on the cell by spin coating method and heated at 110 °C for 10 s to form a perovskite.

In making cavity transfer layer, the cells made with spiro-OMeTAD material for the cavity transfer layer usually had the highest efficiency. Of course, this substance is very expensive and affects cell stability. In this construction method, Spiro address layer is explained as the cavity transfer layer. Spiro-OMeTAD coated with spin coating device at 4000 rpm for 30 s.

Gold coating was performed by physical vapor deposition (PVD). This coating is a conventional method for coating different materials from the vapor phase that can physically produce thin layers or coatings on the surface of various substrates. The thickness of this layer is 80 nm.

### Identification analysis and determination of efficiency

#### *Investigation of the infrared (FT-IR) spectra of TiO*_*2*_* and ZnO quantum dots and their composites*

**Figure 1:**
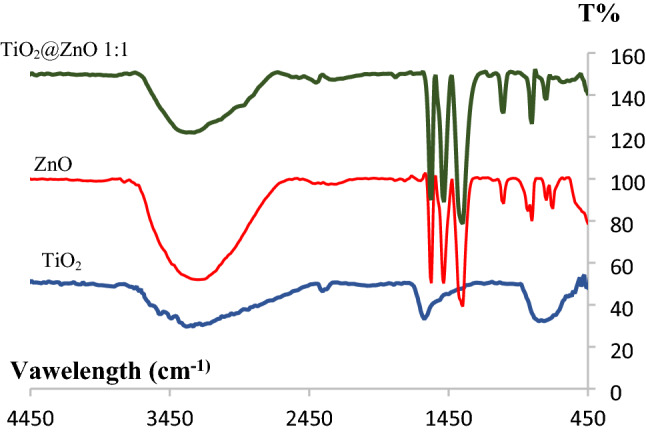
FT-IR spectra of ZnO and TiO_2_ quantum dots and their composites.

Figure 1 shows the infrared spectrum of TiO_2_ and ZnO quantum dots and their composite. Infrared spectrum related to ZnO quantum dots, peak 447 cm^−1^ related to Zn–O vibration, peak 705 cm^−1^ related to Zn–OH, peak 1352 cm^−1^ related to C=O bond Zinc acetate, spectrum 1487 cm^−1^ Related to the C=C bond of zinc acetate, the peak of 1577 cm^−1^ corresponds to the stretching vibration of C–H zinc acetate and the peak of 3294 cm^−1^ corresponds to the O–H bond.

In the case of the infrared spectrum of the wide peak TiO_2_ quantum dots observed at 3000–3400 cm^−1^, it is related to the stretching vibration of the hydroxyl group (–OH), which represents water as moisture. The peak observed in 1627 cm^−1^ is related to the stretching C=O of titanium carboxylate, which is due to the presence of titanium isopropoxide and ethanol as a precursor. The weak peak observed in 1047 cm^−1^ belongs to the Ti–O–C group and the peak observed in 474 cm^−1^ and 800 cm^−1^ is due to the presence of stretching Ti–O in TiO_2_.

#### Investigation of FE-SEM images of TiO_2_ and ZnO quantum dots

**Figure 2:**
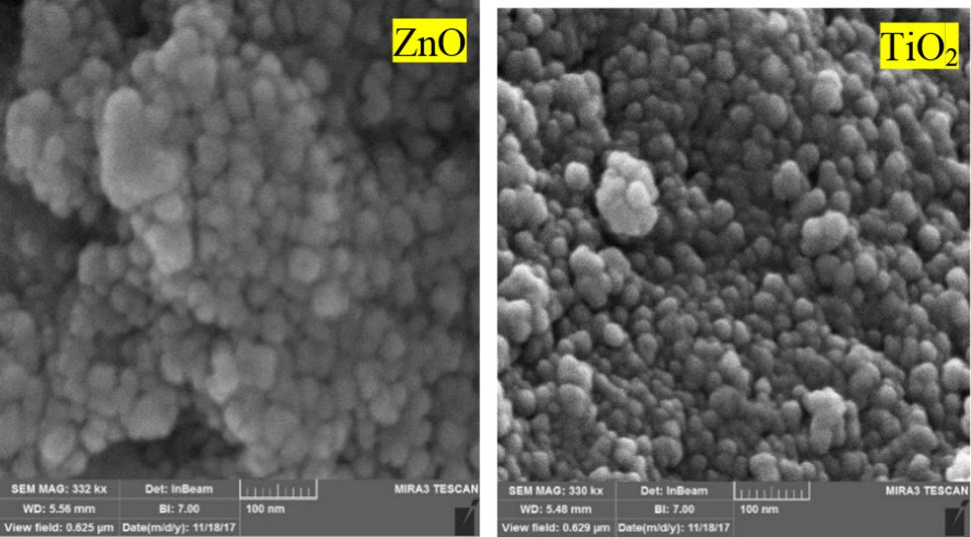
FE-SEM of TiO_2_ quantum dots and ZnO quantum dots.

FE-SEM images show TiO_2_ and ZnO quantum dots, particle size distribution, and particle morphology. As shown in Fig. 2, TiO_2_ quantum dots have a spherical morphology and have a particle size distribution between 6 and 8 nm. The particles are slightly agglomerated, resulting from the agnomerization of small quantum dots. In the case of ZnO quantum dots, it is also observed that the particles have a spherical morphology and have a particle size distribution between 5 and 9 nm. To obtain the average particle size of ZnO and TiO2 in TEM images, Digimizer software was used.

#### Investigation of TEM images of TiO_2_ and ZnO quantum dots and TiO_2_@ZnO composite

**Figure 3:**
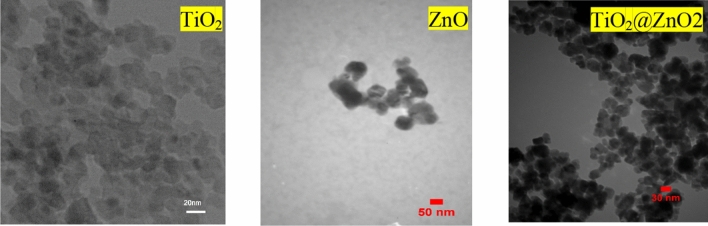
TEM of TiO_2_ quantum dots, ZnO quantum dots and TiO_2_@ZnO composite.

Figure 3 shows the TEM images of TiO_2_ and ZnO quantum dots and their composite. The average particle size of TiO_2_ quantum dots is about 7 nm. This indicates that different sizes of irregular shapes of TiO_2_ quantum dots have grown in large quantities in the agnomere state and the average of ZnO quantum dots is 6 nm. To obtain the average particle size of ZnO, TiO_2_, and TiO_2_@ZnO in FE-SEM images, Digimizer software was used.

#### *XRD characterization of TiO*_*2*_* and ZnO quantum dots and TiO*_*2*_*@ZnO composite*

**Figure 4:**
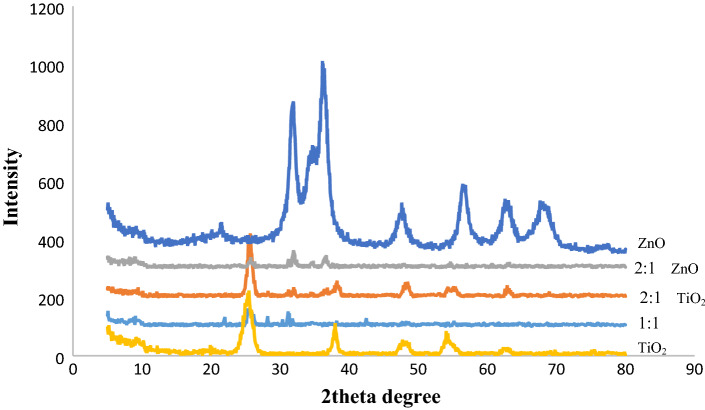
XRD pattern of TiO_2_ quantum dots, ZnO quantum dots and TiO_2_@ZnO composite.

As shown in Fig. 4, diffraction peaks at 25.3°, 38.0°, 48.1°, 54.2°, 55.1°, 62.8°, 68.9°, 70.4° and 75.3° (2θ) marked in XRD pattern are well indexed with crystal planes of anatase (101), (004), (200), (105), (211), (204), (116), (220) and (107) indices of TiO_2_, respectively. This observation confirms the existence of anatase phase which matches well with the reference card (JCPDS-#21-1272)^31,36,37^.

Diffraction peaks at 31.68°, 34.35°, 36.09°, 47.36°, 56.48°, 62.70°, 66.23°, 67.87°, 68.99° and 76.77° marked in XRD pattern with crystallographic pages (1 0 0), (0 0 2), (1 0 1), (1 0 2), (1 1 0), (1 0 3), (1 1 2) and (2 0 1) are well indexed vortex phase of ZnO respectively which is well compatible with the reference card (JCPDS No 36-1451)^7^. In Fig. 4, there are some noises. These noises may indicate some impurities, a very short scan time in XRD, the XRD device is not calibrated or even the device is old.

#### *Investigation of I–V diagram of Perovskite solar cell in 2:1 ratio of TiO*_*2*_*@ZnO composite*

**Figure 5:**
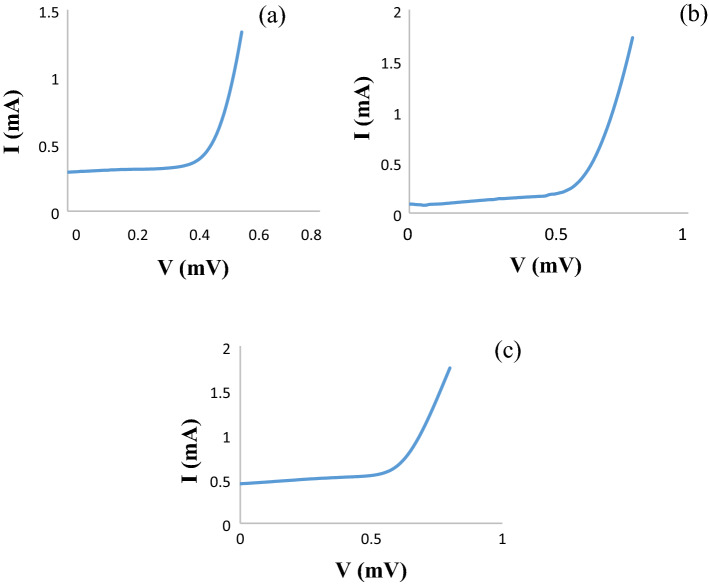
I–V graph (**a**) 1:1 TiO_2_@ZnO, (**b**) 2:1 ratio of TiO_2_@ZnO, (**c**) 2:1 ratio ZnO@TiO_2_ composite.

Current–voltage analysis is the first and most basic analysis of a solar cell. In this analysis, cell efficiency as well as open circuit voltage, short circuit current and cell filling factor are determined. Three perovskite solar cells for each ratio were fabricated and three tests were taken from each to obtain the best performance. The best yield for 2:1 TiO_2_:ZnO ratio was 5.21%, for 1:1 TiO_2_:ZnO was 2.3 and for 2:1 ZnO@TiO_2_ was 3.7. Results of Fig. 5 were obtained from the calculation of current and voltage according to the calculations performed.

#### Investigation of the bandgap of TiO_2_ and ZnO quantum dots and their composites

**Figure 6:**
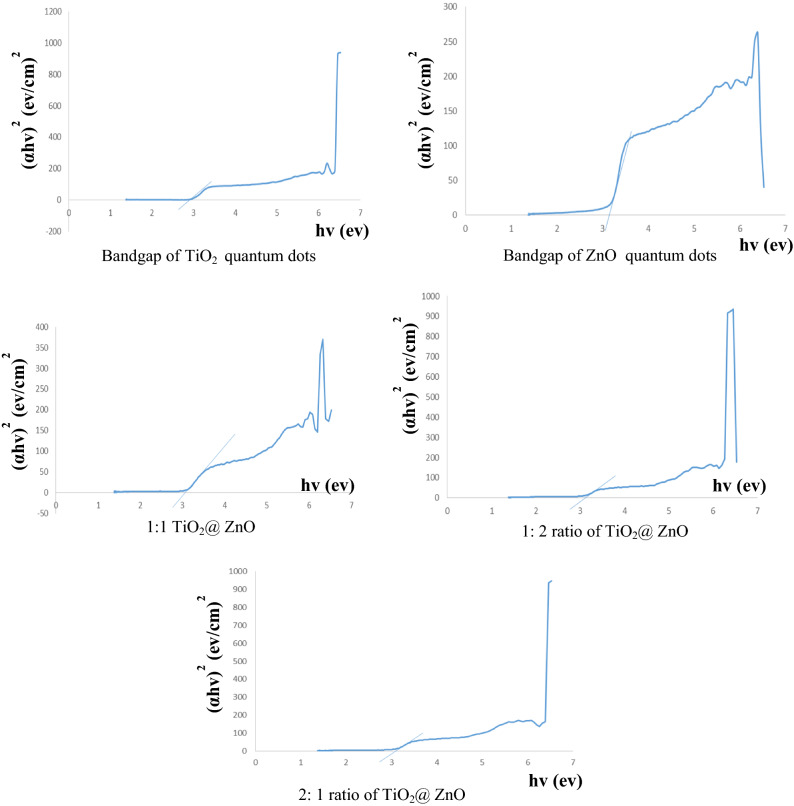
Bandgap of TiO_2_ and ZnO quantum dots and their composites.

According to the diagrams Fig. 6, the bandgap of titanium dioxide quantum dots is 3 electron volts and the bandgap of Zn oxide quantum dots is 3.2 electron volts. The bandgap of different ratios of TiO_2_ @ ZnO composite is about 3/1 electronvolt.

## Discussion and conclusion

Due to the growing energy consumption, limited current energy production resources, increased productivity of solar-powered equipment, the simplicity of installation, commissioning and use of equipment and the low cost of equipment with solar energy the use of solar energy is becoming more widespread day by day. Perovskite solar cells are of particular importance because of the high efficiencies they have achieved in recent years. The use of quantum dots has also played an important role in the efficiency of these cells and their efficiency. The TiO_2_@ZnO composite was synthesized for the first time and the application of this composite with different ratios of TiO_2_ and ZnO quantum points was investigated and the efficiency was determined. The efficiency of the cell was reported to be about 5% in a solar cell made of 2:1 ratio TiO_2_@ZnO composite, which indicates that the presence of titanium oxide is better than zink oxide and increases the efficiency and movement of electrons**.** There are other perovskite solar cells that have higher efficiency about 22% and higher, but certainly, our research on this new hybrid could be a good infrastructure for further studies to improve the efficiency of perovskite solar cells with the compounds of this hybrid. The whole efficiency of this solar cell, can be increased by optimizing the conditions or by composing with other materials. In order to increase the efficiency, it is possible to optimize the construction conditions of the perovskite solar cell (in terms of layering, layer thickness, rotational layering time, etc.); Improving the stability of ZnO quantum dots by composing TiO_2_@ZnO composites with other materials (such as PbS and materials that affect the efficiency of the solar cell) are other ways to increase efficiency. Also, the use of TiO_2_@ZnO composite in core–shell composites, the use of TiO_2_@ZnO composite in multiple (tandem solar cells), and finally locating and optimizing the establishment of solar cell fields are other ways to increase efficiency.

All these results are the result of the work of the research team in the laboratory of Iran University of Science and Technology. Mentioning the sources has been to match the results of our experiments with the correct scientific sources.

## Data Availability

All data generated or analysed during this study are included in this published article.
